# Reduced prevalence of drug-related problems in psychiatric inpatients after implementation of a pharmacist-supported computerized physician order entry system - a retrospective cohort study

**DOI:** 10.3389/fpsyt.2024.1304844

**Published:** 2024-04-09

**Authors:** Katharina Wien, Julia Thern, Anika Neubert, Britta-Lena Matthiessen, Stefan Borgwardt

**Affiliations:** ^1^ Department of Hospital Pharmacy, Universitätsklinikum Schleswig-Holstein, Lübeck, Germany; ^2^ Department of Psychiatry and Psychotherapy, Center for Integrative Psychiatry, Universitätsklinikum Schleswig-Holstein, Lübeck, Germany; ^3^ Department of Psychiatry and Psychotherapy, Center of Brain, Behavior and Metabolism, Universität zu Lübeck, Lübeck, Germany

**Keywords:** computerized physician order entry system, clinical decision support system, medication review, medication prescription, drug-related problems, mental health

## Abstract

**Introduction:**

In 2021, a computerized physician order entry (CPOE) system with an integrated clinical decision support system (CDSS) was implemented at a tertiary care center for the treatment of mental health conditions in Lübeck, Germany. To date, no study has been reported on the types and prevalence of drug-related problems (DRPs) before and after CPOE implementation in a psychiatric inpatient setting. The aim of this retrospective before-and-after cohort study was to investigate whether the implementation of a CPOE system with CDSS accompanied by the introduction of regular medication plausibility checks by a pharmacist led to a decrease of DRPs during hospitalization and unsolved DRPs at discharge in psychiatric inpatients.

**Methods:**

Medication charts and electronic patient records of 54 patients before (cohort I) and 65 patients after (cohort II) CPOE implementation were reviewed retrospectively by a clinical pharmacist. All identified DRPs were collected and classified based on ‘The PCNE Classification V9.1’, the German database DokuPIK, and the ‘NCC MERP Taxonomy of Medication Errors’.

**Results:**

325 DRPs were identified in 54 patients with a mean of 6 DRPs per patient and 151.9 DRPs per 1000 patient days in cohort I. In cohort II, 214 DRPs were identified in 65 patients with a mean of 3.3 DRPs per patient and 81.3 DRPs per 1000 patient days. The odds of having a DRP were significantly lower in cohort II (OR=0.545, 95% CI 0.412-0.721, p*<*0.001). The most frequent DRP in cohort I was an erroneous prescription (n=113, 34.8%), which was significantly reduced in cohort II (n=12, 5.6%, p*<*0.001). During the retrospective in-depth review, more DRPs were identified than during the daily plausibility analyses. At hospital discharge, patients had significantly less unsolved DRPs in cohort II than in cohort I.

**Discussion:**

The implementation of a CPOE system with an integrated CDSS reduced the overall prevalence of DRPs, especially of prescription errors, and led to a smaller rate of unsolved DRPs in psychiatric inpatients at hospital discharge. Not all DRPs were found by plausibility analyses based on the medication charts. A more interactive and interdisciplinary patient-oriented approach might result in the resolution of more DRPs.

## 1 Introduction

Patients with mental disorders often require psychopharmacological treatment and are often prescribed combinations of psychopharmacologically active drugs, such as antidepressants, antipsychotics, mood stabilizers, anxiolytics, and hypnotics. Additionally, many psychiatric patients present themselves with somatic comorbidities which require pharmacological treatment. Prescription data from public health insurance providers in Germany show that outpatients were prescribed more than two billion daily defined doses (DDD) of psychotropic drugs in 2021, most of which were antidepressants (1.7 billion DDD, 79% of psychotropic drug prescriptions including tranquillizers, antipsychotics and antidepressants) (1). In a big retrospective study conducted in eight psychiatric hospitals in Germany including 14,418 inpatient cases, 31% of cases received at least five drugs simultaneously (polypharmacy) with a mean of 3.7 drugs daily (1.7 psychotropic drugs, 2.0 others) (2). In addition, due to the widespread anticholinergic properties of psychotropic drugs, especially antipsychotics (3–5), researchers have pointed out the need for interventions to reduce the prescription frequency of anticholinergic drugs and their assessment regarding their impact on clinical risks, especially in patients older than 65 years (6, 7). In a big pharmacovigilance study in ten German psychiatric hospitals, 35.4% of 27,396 patients were prescribed 1-4 anticholinergic drugs (6). These results show the need to further improve pharmacotherapy in psychiatric inpatients using tailored interventions with a special regard to the prescription of anticholinergic drugs. A validated score to assess a patient’s anticholinergic burden (ACB) due to medication prescribed in Germany is the anticholinergic burden score for German prescribers (ACB score) introduced by Kiesel et al. (8).

Polypharmacy, as a common practice in psychiatry (9), increases the risk for drug-related problems including adverse drug reactions (10, 11). The Pharmaceutical Care Network Europe (PCNE) defines a drug-related problem (DRP) as an event or circumstance involving drug therapy that actually or potentially interferes with desired health outcomes (12). One strategy for the solution and prevention of DRPs is the conduction of medication reviews in which clinical pharmacists evaluate patients’ medicines, detect DRPs and recommend interventions with the aim of optimizing medicines use and improving health outcomes (13). Structured medication reviews by clinical pharmacists may reduce polypharmacy, DRPs, medication errors (MEs), and subsequently decrease the rate of adverse drug events (ADEs) including adverse drug reactions (ADR) (14).

Medication reviews in inpatient settings usually include the evaluation of patients’ medication charts. Traditionally, medication of hospitalized patients has been prescribed by his or her physician on handwritten paper charts without a direct clinical decision support system (CDSS) checking for potential errors or risky combinations. Some possible DRPs are directly linked to the paper-based prescription process, e.g. the readability of a prescription. It has been demonstrated that the digitalization of medication charts using medication software and computerized physician order entry (CPOE) systems may help to avoid transmission errors and may simplify the examination of medications regarding existing drug interactions and MEs (15–17). However, the use of medication software may also lead to errors, e.g. due to delayed documentation of drug administration or difficulties in the correct use of the CPOE system, especially if nonstandard specifications are prescribed (18).

Previously published studies examined the implementation of a CPOE system with CDSS in two German general hospitals with a focus on formal criteria of prescription quality (e.g. readability, completeness and clarity, documentation of medication at admission, documentation of allergies) (17, 19). Both studies reported a decrease in formal prescription error rates after implementation of the digital medication charts but did not study DRPs in general. Schaefer et al. (17) also stated that CPOE implementation led to a higher satisfaction of physicians with the documentation system and to a decrease in time needed for drug prescription. Furthermore, the introduction of CPOE and CDSS has been studied extensively in the international literature. A systematic review on interventions to reduce medication errors in adult medical and surgical settings included multiple studies indicating the beneficial effects of CPOE implementation on error rates, such as prescription errors including documentation discrepancies and contraindications (20). Another systematic review on the effect of CPOE and CDSS on ME and ADE rates reported a 71% overall reduction of prescription errors after CPOE implementation (21). However, there were no significant differences in rates of validation, dispensing, and administration errors with CPOE versus manual prescribing (21). One study included in both reviews used pharmacist order checking in addition to CPOE implementation (22) in an orthopaedic surgery unit and found a significant reduction in prescription and administration errors but did not assess the prevalence of content-related medication errors such as drug-drug interactions (DDIs).

To date, no systematic before and after study has been reported on the types, prevalence and severity of DRPs before and after CPOE implementation in a psychiatric inpatient setting. In 2021, a CPOE system with an integrated CDSS was implemented at a tertiary care center for the treatment of mental health conditions in Lübeck, Germany. Since then, the medication prescriptions and all documentations concerning the patients’ medications have been recorded in digital medication charts instead of the paper charts used before. The CDSS is supposed to support physicians in the process of drug prescription and facilitate medication review by hospital pharmacists.

The aim of this retrospective before-and-after cohort study was to investigate whether the implementation of a CPOE system with CDSS accompanied by the introduction of regular medication plausibility checks by a hospital pharmacist leads to a decrease of DRPs during hospital stay and less unsolved DRPs at discharge in psychiatric inpatients. Furthermore, we aimed to assess the types and prevalence of DRPs before and after CPOE implementation.

## 2 Materials and methods

### 2.1 Setting

This study was conducted at the Center for Integrative Psychiatry (ZIP gGmbH) in Lübeck, a subsidiary of University Medical Center Schleswig-Holstein in Lübeck, Germany, and a tertiary care center for the treatment of mental health conditions. The retrospective study was approved by the responsible Ethics Committee of the Medical Faculty at the Universität zu Lübeck (22–094). The online satisfaction survey after implementation of the CPOE system with CDSS among nurses and physicians at the psychiatric facility was approved by the staff council of the Center for Integrative Psychiatry.

Between April 2021 and November 2021, a CPOE system including an integrated advanced CDSS (Meona^®^, release 85.559 a6) was implemented in the electronic health record (Orbis^®^, Dedalus Healthcare Systems Group, Release MR08043600, HF01 ORBIS 080436) on all inpatient (n=7) and day units (n=2) at the Center for Integrative Psychiatry. The CDSS in Meona^®^ includes data from different sources listed in **Supplementary Table S1** in the **Supplementary Material**.

Together with the implementation of the CPOE system with an integrated CDSS, a clinical pharmacist service consisting of the pharmaceutical validation of prescribed medication in the digital medication charts was introduced at the psychiatric wards and day hospital units. The validation process comprises checking prescribed dosages using SmPCs and current guidelines, need for dosage adjustments due to reduced organ functions, especially renal function, potentially clinically relevant drug-drug interactions, and existence of contraindications. Prescriptions containing clinically relevant DRPs are left unvalidated by the clinical pharmacist. In these cases, the pharmacist writes a note to the treating psychiatrist or psychiatric resident in the patient’s medication chart under the prescribed medication on the current day containing a summary of the problem and a proposal for an intervention. If the pharmacist estimates the DRP to be severe and if the physician does not respond to the proposed intervention on the same day, the pharmacist calls him or her on the phone to discuss the problem. During the study period, the pharmaceutical validation was conducted by a clinical pharmacist daily from Monday until Friday or at least three times per week.

### 2.2 Study design

**Figure 1:**
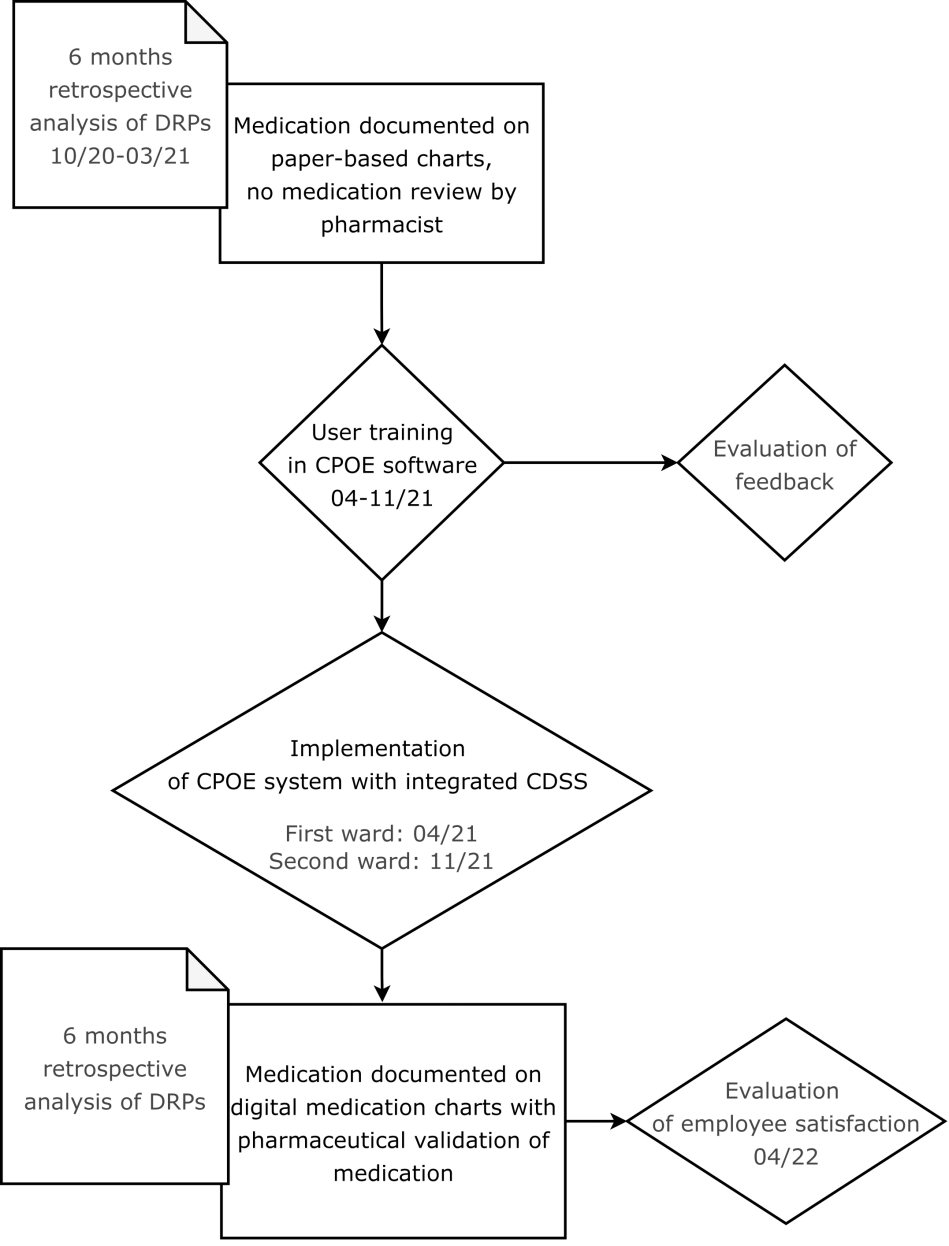
Flow chart showing the process of the CPOE implementation and its evaluation.

In this retrospective before-and-after study, the benefit of the implementation of a CPOE system with integrated CDSS accompanied by the introduction of regular pharmaceutical validation on the rate of DRPs and their resolution was assessed. The study process is illustrated in a flow chart in **Figure 1**.

Since ADEs have been reported to be responsible for 13.6% of patient admissions to a mental health center (23), a focus was set on content-related prescription quality. As the number of DRPs per patient increases per additional drug prescribed on admission (10), the total number of active ingredients (Total Drug Burden, TDB) and the total number of drugs with anticholinergic properties (TDB_AC_) were documented on the first day after hospital admission. Additionally, the ACB score was calculated for all drugs prescribed on this day using the list published by Kiesel et al. (8). As anticholinergic effects of drugs are dose-dependent, the ACB scores identified in this study were analyzed in consideration of the prescribed daily doses. Using the drug burden index for medications with anticholinergic effects (DBI_AC_, Equation 1) (24), the prescribed doses of the corresponding drugs were documented together with the ACB scores in order to estimate the patient-specific risk for the occurrence of anticholinergic side effects. The second inpatient day as the first full inpatient day was chosen for the assessment of the scores described above to ensure comparability of the two study cohorts before and after CPOE implementation.


(1) $$DBI_{AC}=\sum \frac{D}{\delta +D}$$

DBI_AC_: Drug burden index for drugs with anticholinergic effects
D: Daily prescribed dose
*δ*: Recommended minimum daily dose

Within the framework of this study, drugs prescribed only as needed (pro re nata, PRN) were excluded from the calculation of DBI_AC_. For the German drug market, within the COFRAIL-study (25), funded by the innovation funds, grant number 01VSF17053, the COFRAIL-study group has created the COFRAIL-Drug Burden List containing a total of 197 anticholinergic and/or sedative drugs and their respective minimum daily doses. The COFRAIL-Drug Burden List has not been published but was accessed with permission of the study team. The minimum daily doses for anticholinergic drugs were used as reference for the calculation of the DBI_AC_ in this study. If a drug listed in Kiesel et al. (8) was not included in the COFRAIL-Drug Burden List, the minimum daily dose recommended in the corresponding SmPC was used for DBI_AC_-calculation.

Before the implementation of the CPOE system, prescriptions and subsequent changes were documented on paper charts by physicians, usually residents, or by nurses following verbal instructions by a physician. Since the introduction of the CPOE system, the prescription process has been a physician’s responsibility. The nurses, however, are still responsible for the documentation of medication dispensing and administration.

In the present study, a clinical pharmacist retrospectively conducted intermediate medication reviews, type 2b, based on medication history and medical information according to the PCNE classification of medication reviews (13) of at least 50 paper-based medication charts before (cohort I) and 50 digital medication charts after CPOE implementation (cohort II). For practical reasons, the medication charts of patients in cohort II were mostly analyzed prior to those of patients in cohort I. To ensure comparability of results based on the same CDSS in both cohorts, the prescribed medication per patient in the pre-implementation cohort was transferred from the respective paper charts into a blank digital chart in the CPOE system by the clinical pharmacists conducting the medication reviews. This implies that all warnings by the CDSS were included in both cohorts with the exception of warnings related to laboratory data, sex and age in the pre-implementation cohort. All formal and content-related prescription errors, DRPs, pharmaceutical interventions documented in the digital charts, status of DRP at time of discharge, and patient outcome, as well as TDB, TDB_AC_, ACB score and DBI_AC_ on the first day after hospital admission were collected pseudonymously in an SQL database.

#### 2.2.1 Study size

This was the first before-and-after cohort study on the rates of DRPs in a psychiatric setting after CPOE implementation. One study estimated the adjusted effect of a pharmaceutical intervention on the rates of unsolved DRPs per patient in a psychiatric inpatient setting to be 1.82 (95% CI 1.52-2.14) less unsolved DRPs per patient (26). In consideration of Wolf et al. (26) and Jungreithmayr et al. (19), an effect size w of 1.309 was calculated in G*Power 3.1.9.7 (27). In order to achieve 80% power for an effect size of 1.309 with a two-tailed significance level of 0.05, only 5 patients were required according to a Chi^2^-Goodness-of-fit test. As the Chi^2^-test is only recommended for study sizes bigger than 50 patients, a sample size of at least 50 patients in cohort I and cohort II, respectively, was defined. Due to the retrospective design of the study without follow-up, no drop-outs were expected.

### 2.3 Subjects/Study population

Patients on two wards specialized in depression, anxiety, and obsessive-compulsive disorder with a maximum capacity of 60 beds were screened for study inclusion. The study period included six months from October 1^st^ 2020 until March 31^st^ 2021 for the analysis of paper charts (cohort I) and six months after the implementation of the CPOE system (cohort II) from April 15^th^ 2021 until October 15^th^ 2021 for the first ward and from November 3^rd^ 2021 until May 3^rd^ 2022 for the second ward. While on the first ward, CPOE implementation was completed in April 2021, it was only accomplished in November 2021 on the second study ward. More patients from the first ward than from the second ward were initially included in the post-implementation group. To achieve equal group sizes with a minimum of 25 patients per ward for both cohorts, another 14 patients from the second ward were included in the study.

#### 2.3.1 Inclusion and exclusion criteria

Patients were included if they were admitted to one of the two study wards during the study periods, gave broad consent to the use of their clinical data for research purposes, if they were 18 years or older and if they were prescribed at least one drug during their hospital stay. Patients were excluded from the pre-implementation group if a scan of their paper chart was not or not entirely available in the electronic archive. Additionally, patients were excluded from both groups if medication was first prescribed on the day of hospital discharge or if their only hospital stay was during the CPOE implementation period. Psychiatric patients are often admitted to a psychiatric ward multiple times (28). In order to achieve independent study groups, the same patient was only included in the study once, either in the pre-implementation or in the post-implementation cohort.

### 2.4 Data collection

For both independent groups, the TDB, TDB_AC_, ACB and DBI_AC_ scores were calculated from the prescription data on the respective medication charts of the patients’ second inpatient treatment day by one pharmacist (KW). Demographic data collected included ward, date of admission to and discharge from the ward, date of birth, sex, psychiatric diagnoses (ICD-10 codes F), number of stays at one of the two study wards during the study periods, and whether they had a stay during the CPOE implementation period. Medication reviews were conducted by the same pharmacist (KW) using prescription data from paper or electronic charts, respectively, laboratory data, summary of treatment and discharge medication from the doctors’ letters, and history entries by nurses, physicians, and psychotherapists in the synopsis of electronic patient records. Data from medication charts and electronic patient records were collected pseudonymously in an SQL database by one pharmacist (KW).

### 2.5 Data appraisal

Different systems for the classification of DRPs and MEs have been published and validated in Germany, e.g. The PCNE Classification (29), NCC MERP Taxonomy of Medication Errors (30), PI-Doc (31), and Doku-PIK (32). The PCNE Classification V 9.1 comprises a basic classification with primary domains and respective subdomains for problems, causes, planned interventions, level of acceptance (of interventions), and status of the DRP. Contrary to other classification systems, the PCNE classification contains a domain for rating the outcome of the intervention as “status of the problem” (12) and was therefore chosen as the main classification system for DRPs in this study. Some categories were added based on Doku-PIK (32), for patient outcome on the NCC MERP Taxonomy of Medication Errors (33) and by the study team (IDM-PSY-PHARM), **Supplementary Table S2** in the **Supplementary Material**. DRPs were rated as potential if they could possibly cause harm to the patient but no harm has been detected. If they caused harm to the patient or if they were definite errors such as prescription errors, DRPs were rated as manifest.

Prior to the main study, an inter-rater reliability assessment of five electronic medication charts was carried out by five pharmacists (KW, AW, JT, CR, MW) using the preliminary classification table designed by the study team. Based on the results, the categories “setting” and “involved personnel” were excluded from the final version. Furthermore, the definitions of the TDB_AC_- and the DBI_AC_-scores were clarified: the TDB_AC_-score included the prescribed PRN medication whereas the DBI_AC_-score did not include it.

To ensure rater objectivity in the identification and classification of DRPs in the main study, all DRPs documented by one pharmacist (KW; four years of clinical experience) were checked by a second pharmacist (AN; six years of experience as a licensed pharmacist incl. one year of clinical experience). If the second pharmacist identified further DRPs, she documented them in a separate Microsoft Excel sheet. Differences in documented DRPs and their classifications were then discussed until agreement was achieved. When consensus could not be achieved between the two pharmacists, a third pharmacist (JT; *>*20 years of clinical experience) was consulted to finalize the rating.

### 2.6 Validity assessment

The DRPs documented in this study were not documented independently but searched for in detailed patient files by the two pharmacists. Furthermore, multiple categories could be chosen in the classification, based on the validation of the German classification system DokuPIK (34). Therefore, the concordance level of the two pharmacists to the final rating was calculated to ensure validity of results. The following definition was used for the rater agreement (34) Equation 2:


(2) $$\text{Proportion of rater agreement}=\frac{\text{positive and negative votes concordant with the final rating}}{\text{total votes}}\cdot 100\%$$

### 2.7 Employee satisfaction survey after CPOE implementation

One year after CPOE implementation on the first pilot ward, an employee satisfaction survey was conducted using the hospital’s online survey tool evasys [evasys GmbH, V8.2, Webserver/Datenbank IIS/MySQL (127.0.0.1)] between March 24th 2022 and April 15th 2022. Nurses and physicians working on the psychiatric wards and day units were invited to participate in the online survey via e-mail: all physicians were e-mailed personally, the team leaders (nurses) of all wards and day units were e-mailed in person and asked to forward the survey to their team members (nurses). Additionally, the nurse manager was informed in advance and included in the mailing list. Two reminders were sent to the same mailing list (7 and 18 days after the initial invitation).

### 2.8 Statistical analysis

The study team was consulted by the Institute of Medical Biometry and Statistics at Universität zu Lübeck on the study design in December 2021. As the group allocation was not random but assigned based on the time of hospital admission, the group differences at baseline were assessed using Mann-Whitney-U for the continuous variables (age, DBI_AC_) and Chi-squared or Fisher’s exact test for the nominal or discrete variables (gender, length of hospital stay, count of hospital stays per patient, TDB, TDB_AC_ and ACB score). To estimate the effect of the implementation of the CPOE system with CDSS and medication review by a pharmacist on the primary endpoints, a regression analysis to adjust for group differences at admission was performed. A generalized mixed methods linear model for the negative binomial distribution was computed using Jamovi^®^ (The jamovi project (2021). jamovi. (Version 1.6) [Computer Software]. Retrieved from https://www.jamovi.org) to estimate the intervention’s effect on the rate of DRPs per patient and of unsolved DRPs per patient at discharge. The rates of DRPs per patient and unsolved DRPs served as dependent variables that were compared in two independent groups before (cohort I) and after CPOE implementation (cohort II). Ward and sex were chosen as factors and the following covariates were included in the model: group allocation (cohort I or II), length of hospital stay, and age. The rates of DRPs in both groups were estimated in 95%-confidence intervals.

The rates of the respective classified DRPs, patient outcome, related ATC-groups of causative drugs, mean TDB, TDB_AC_, ACB score, and DBI_AC_ and the results of the employee satisfaction survey were evaluated descriptively.

## 3 Results

### 3.1 Drug-related problems before and after CPOE implementation

#### 3.1.1 Patient characteristics

**Figure 2:**
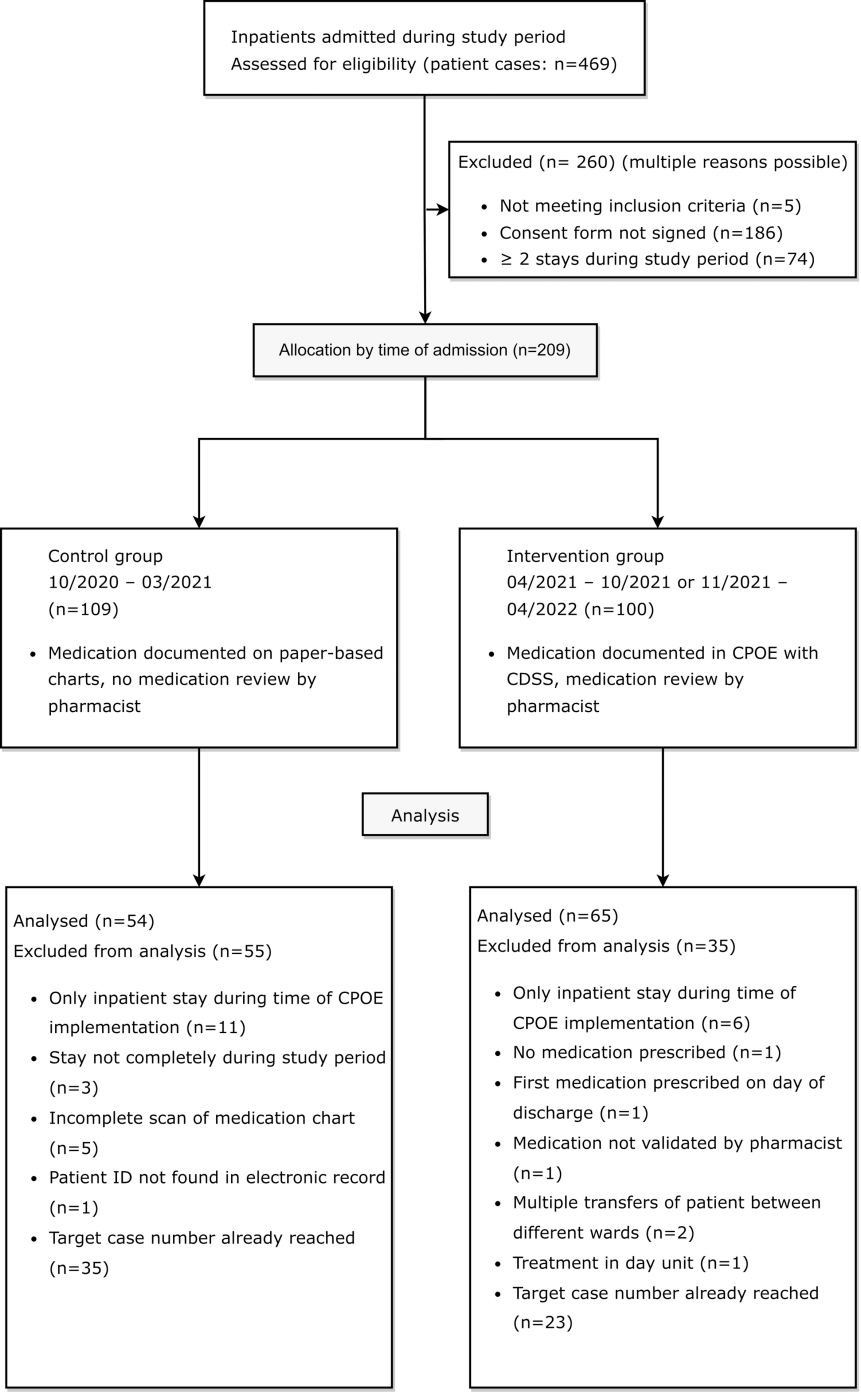
Flow diagram of eligible patients for retrospective medication reviews in the control group before and in the intervention group after CPOE implementation, adapted by the CONSORT 2010 Flow Diagram (35).

The study inclusion and exclusion process is shown in a flow diagram in **Figure 2**.

##### 3.1.1.1 Pre-implementation cohort

Between October 1^st^ 2020 and March 31^st^ 2021, 244 patient cases were treated on the two psychiatric study wards. In 57.8% of these cases (141/244), patients had agreed to the use of their clinical data for research purposes at admission. In 50 of the 141 cases with consent (35.5%), a total of 18 patients were treated on one of the two study wards at least twice during the study period (range: 2-7 inpatient stays). A total of 109 patient cases were eligible for study inclusion in the pre-implementation cohort.

##### 3.1.1.2 Post-implementation cohort

After CPOE implementation on the first ward, between April 19^th^ and October 19^th^ 2021, 106 patient cases were treated on the first ward. 41 out of 106 cases (38.7%) were excluded because patients had not signed the consent form to the use of their clinical data for research purposes (36/106, 34.0%), were not mainly treated on the first ward (2/106, 1.9%), were treated mainly in the day unit (1/106; 0.9%), as pre-inpatient (1/106, 0.9%) or were under 18 years old (1/106, 0.9%). Out of the 65 patient cases meeting inclusion criteria, a total of 13 patients were treated on the ward at least twice during the study period in 33 different cases (range: 2-6 inpatient stays). A total of 45 patient cases from the first ward (45/106, 42.4%) were eligible for study inclusion in the post-implementation cohort. After CPOE implementation on the second ward, between November 3^rd^ and May 3^rd^ 2021, 119 patient cases were treated on the ward specialized in depression, anxiety, and compulsive disorder. 42 out of 119 cases (35.3%) were excluded because patients had not signed the consent form to the use of their clinical data for research purposes. Out of the 77 patient cases meeting inclusion criteria, a total of 13 patients accounted for 35 cases (range: 2-5 inpatient stays). A total of 55 patient cases from the second ward (55/119, 46.2%) were eligible for study inclusion in the post-implementation cohort.

Out of all included patients, 21 were treated on one of the two study wards in both the pre-implementation and the post-implementation period. Three of these patients were excluded from the study during the data collection process due to an incomplete scan of the paper-based medication chart. To achieve comparability between groups, ten and eight random patients with multiple stays during both study periods were included in the pre- and post-implementation group.

20 patients in cohort I (control group) and twelve patients in cohort II (intervention group) were excluded for different reasons listed in **Figure 2**. In accordance with the pre-defined goal of at least 50 included patients per group in the study protocol, the paper-based medication charts of 27 patients from each of the two evaluated wards were included in the study. In the post-implementation group, the digital medication charts of 38 patients from the first ward and of 27 patients from the second ward were included in the study. In total, DRPs in 54 patients in the pre-implementation group (cohort I) and 65 patients in the post-implementation group (cohort II) were retrospectively analyzed and documented for a total of 2140 and 2633 inpatient days, respectively.

In most demographic categories, there were no significant group differences at baseline. Patients had a mean age of 46.0 (SD 14.9) years and 44.5 (SD 17) years in cohorts I and II (p=0.6) and more than 60% were women (p=0.87). More than three quarters of all patients had a diagnosis of depression in both groups (43/54, 80% and 50/65, 77%, p=0.82), the most frequent diagnosis being F33.2: Recurrent depressive disorder, current episode severe without psychotic symptoms (33/54, 61% and 32/65, 49%, p=0.20). However, the patients in cohort II stayed in hospital significantly longer (mean 40.5, SD 23.4 days) than the patients in cohort I (mean 39.6, SD 20.3 days, p*<*0.001) and there were significantly more patients with a diagnosis of a personality or behavioral disorder in cohort I (18/54, 33%) than in cohort II (7/65, 11%, p=0.003). For detailed patient characteristics in the study groups before and after CPOE implementation, see **Table 1**.

**Table 1:** Patient characteristics in the study groups before and after CPOE implementation.

Variable	Cohort I (n= 54)	Cohort II (n= 65)	p-value
Mean age [years] (SD)	46.0 (14.9)	44.5 (17)	0.60^l^
Sex: Female	34 (63%)	40 (62%)	0.87^k^
Mean length of hospital stay [days] (SD)	39.6 (20.3)	40.5 (23.4)	*<*0.001^j^
Mean count of hospital stays (SD)	1.7 (1.5)	1.4 (1)	0.14^j^
Ward I (%)	27 (50%)	38 (58%)	0.46^j^
Most frequent diagnosis No. of patients	F33.2 33 (61%)	F33.2 32 (49%)	0.20^j^
Psychiatric diagnoses^a^			
Depression^b^	43 (80%)	50 (77%)	0.82^j^
Dysthymia^c^	7 (13%)	6 (9%)	0.56^j^
Neurotic, stress-related, somatoform disorders^d^	26 (48%)	31 (48%)	*>*0.99^j^
Personality and behavioral disorders^e^	18 (33%)	7 (11%)	0.003^j^
Mental/behavioral disorder due to psychoactive substance use^f^	6 (11%)	10 (15%)	0.59^j^
Bipolar affective disorder^g^	2 (4%)	1 (2%)	0.59^j^
Schizophrenia or schizophreniform disorder^h^	1 (2%)	3 (5%)	0.62^j^
Other psychiatric disorders^i^	7 (13%)	9 (14%)	*>*0.99^j^

Cohort I, Pre-implementation cohort; Cohort II, Post-implementation cohort.

ICD-10 International Statistical Classification of Diseases and Related Health Problems 10th Revision; ^a^Patients could have more than one diagnosis; ^b^ICD-10 F32, F33; ^c^ICD-10 F34.1; ^d^ICD-10 F40, F41, F42, F43, F44, F45; ^e^ICD-10 F60, F61, F62, F63; ^f^ICD-10 F10, F11, F12, F13, F15, F17; ^g^ICD-10 F31; ^h^ICD-10 F20, F22, F25, F29; ^i^ICD-10 F05, F06, F07, F50, F51, F53, F70, F84, F90, F95, G30; ^j^Fisher’s exact test; ^k^Chi-square-test; ^l^Mann-Whitney-U-test.

A list of the ten most documented diagnoses as ICD-10 codes (36) and their frequencies in each study group can be found in the **Supplementary Material** (**Supplementary Table S3**).

In addition, the mean scores on the second inpatient day for TDB, TDB_AC_, ACB and DBI_AC_ for each study group are shown in **Table 2**. None of the mean scores were significantly different at baseline between the two cohorts. On the second inpatient day, patients were prescribed a mean of 4-5 different drugs (TDB: 4.8, SD 3 and 4.8, SD 3.6, p=0.91) with a mean of two anticholinergic drugs (TDB_AC_: 2.2, SD 1.4 and 1.9, SD 1.2, p=0.41). The mean ACB-score was not significantly lower in cohort II (3, SD 2 and 2.4, SD 1.4, p=0.27) and the mean DBI_AC_ was similar (1.11, SD 0.73 and 1.11, SD 0.82, p=0.75).

**Table 2:** Mean scores for TDB, TDB_AC_, ACB and DBI_AC_ in the study groups before and after CPOE implementation.

Score	Cohort I	Cohort II	p-value
TDB (SD)	4.8 (3)	4.8 (3.6)	0.91^a^
TDB_AC_ (SD)	2.2 (1.4)	1.9 (1.2)	0.41^a^
ACB (SD)	3 (2)	2.4 (1.4)	0.27^a^
DBI_AC_ (SD)	1.11 (0.73)	1.11 (0.82)	0.75^b^

Cohort I, Pre-implementation cohort; Cohort II, Post-implementation cohort ^a^Fisher’s exact test. ^b^Mann-Whitney-U-test.

#### 3.1.2 Validity assessment as level of concordance

In all patients included in this study, a total of 535 DRPs were documented by the first author (KW). After discussion with the second pharmacist (AN) and in case of disagreement between KW and AN with the third pharmacist (JT), nine DRPs were excluded because they did not meet the definition of a DRP. Another 13 DRPs found by the second pharmacist (AN) were added to the data set. Finally, a total number of 539 DRPs with their respective categorizations were included in the final analysis. Overall concordance between KW and AN for all categories combined was 98% (range: 96-100%).

#### 3.1.3 Rate of DRPs per 1000 patient days

In cohort I, 325 DRPs were documented in 2140 inpatient days with a mean of 6.0 DRPs (SD 4.7) per patient and at least one DRP in 53 (98%) of the patients (range: 0-21 DRPs per patient). Thus, the overall prevalence of DRPs in the pre-implementation cohort was 151.9 DRPs per 1000 patient days. In cohort II, 214 DRPs were documented in 2633 inpatient days with a mean of 3.3 DRPs (SD 3.2) per patient and at least one DRP in 56 (86%) patients (range: 0-16 DRPs per patient). Therefore, the overall prevalence of DRPs in the post-implementation cohort was 81.3 DRPs per 1000 patient days. In the negative binomial generalized mixed methods linear model, it was calculated that the odds ratio (OR) to experience a DRP was 0.545 in cohort II compared to cohort I (OR=0.545, 95% CI 0.412-0.721, p*<*0.001, Pearson’s r=-0.326). The patients’ age (OR=1.015 per year, 95% CI 1.006-1.024, p*<*0.001) and length of stay on the study wards (OR=1.012 per inpatient day, 95% CI 1.005-1.019, p*<*0.001) were significantly associated with the number of DRPs. Ward affiliation (OR=1.29 on ward I compared to ward II, 95% CI 0.96-1.73, p=0.09) and gender (OR=1.072 for females compared to males, 95% CI 0.803-1.431, p=0.64) did not result in any significant differences in the risk to experience a DRP.

#### 3.1.4 Analysis of classification of DRPs

The most frequent problems and causes, patient outcome, and frequencies of DRPs in both cohorts are shown in **Table 3**. On average, 1.6 (SD 1.1) and 1.6 (SD 0.86) drugs were involved per DRP in cohort I and cohort II. 207 (63.7%) of all DRPs in cohort I and 122 (57%) DRPs in cohort II did not result in any harm to the patient and did not require further monitoring (NCC MERP Patient Outcome Categories A-C). The most frequent cause of DRPs in cohort I was an incomplete or erroneous prescription (34.8% of all DRPs) which was significantly reduced after CPOE implementation (5.6%, p*<*0.001), e.g. haloperidol prescribed 5 mg as PRN medication without a dosage form in cohort I.

**Table 3:** Frequency and classification of DRPs in the study groups before and after CPOE implementation.

	Cohort I	Cohort II	p-value
DRPs overall	325	214	*<*0.001^c^
No. of patients with ≥ 1 DRP	53 (98%)	56 (86%)	0.02^b^
DRPs per patient (SD)	6 (4.7)	3.3 (3.2)	*<*0.001^c^
DRPs per 1000 patient days (SD)	151.9 (118.0)	81.3 (79.1)	*<*0.001^c^
Mean no. of drugs per DRP (SD)	1.6 (1.1)	1.6 (0.9)	0.16^b^
Most frequent DRP (%)	Treatment safety: 193 (59.4%) (Potential) ADR: 141 (43.4%)	Treatment safety: 138 (64.5%) (Potential) ADR: 118 (55.1%)	0.24^b^ 0.008^b^
Most frequent domain of cause (%)	Drug selection: 128 (39.4%)	Drug selection: 111 (51.9%)	0.005^b^
Most frequent cause in cohort I (%)	Erroneous prescription: 113 (34.8%)	Erroneous prescription: 12 (5.6%)	*<*0.001^b^
Most frequent cause in cohort II (%)	Interaction^d^: 100 (30.8%)	Interaction^d^: 75 (35%)	0.30^b^
Manifest problem (%)	172 (52.9%)	86 (40.2%)	0.004^c^
Potential problem (%)	153 (47.1%)	128 (59.8%)	0.004^c^
Patient Outcome (%)	A^a^: 122 (37.5%)	A^a^: 71 (33.2%)	0.31^b^
Totally solved DRPs at discharge (%)	119 (36.6%)	76 (35.5%)	0.86^b^
Unsolved DRPs at discharge (per patient)	192 (3.6 SD 2.93)	130 (2 SD 2.1)	*<*0.001^c^

Cohort I, Pre-implementation cohort; Cohort II, Post-implementation cohort; ^a^NCC MERP 31.1 Category A: Circumstances or events that have the capacity to cause error; ^b^Fisher’s exact test; ^c^Chi-square-test; ^d^PCNE V9.1, C1.3: Inappropriate combination of drugs, or drugs and herbal medications, or drugs and dietary supplements.

In both cohorts, drug interactions caused a large proportion of DRPs (30.8% in cohort I and 35.1% in cohort II, p=0.303), e.g. clomipramine newly prescribed in a patient in cohort II on treatment with 20 mg citalopram, pharmacist advised against overlapping intake due to increased risk for serotonin syndrome. While problems causing temporary harm to the patient requiring interventions (NCC MERP Patient Outcome category E) occurred in both cohorts (10.5% in cohort I an 12.6% in cohort II), two errors with temporary harm to the patient requiring prolonged hospitalization (NCC MERP Patient Outcome category F) and two errors requiring an intervention to sustain life (NCC MERP Patient Outcome category H) only occurred in cohort I (**Table 4**). However, the two DRPs causing an intervention to sustain life were both documented for the same patient who experienced a neuroleptic malignant syndrome after a combination therapy of multiple antidopaminergic and several sedative drugs. Overall, after CPOE-implementation, 27 manifest DRPs resulting in at least temporary harm to the patient (NCC MERP categories E-I) occurred in 65 patients with a mean of 0.42 per patient while there were 38 such DRPs in 54 patients (mean: 0.70 per patient) before CPOE-implementation. Further examples of manifest and potential DRPs are given in **Supplementary Table S4** for cohort I and in **Supplementary Table S5** for cohort II in the **Supplementary Material**.

**Table 4:** Absolute and relative frequencies of patient outcome categories according to the NCC MERP taxonomy of Medication Errors for DRPs in the study groups before and after CPOE implementation.

Patient Outcome	Cohort I [n=325]	Cohort II [n=214]
A	122 (37.54%%)	71 (33.18%)
B	8 (2.46%)	7 (3.27%)
C, medication not administered	7 (2.15%%)	4 (1.87%)
C, medication administered	70 (21.54%)	40 (18.69%)
D	80 (24.62%)	62 (28.97%)
E	34 (10.46%)	27 (12.62%)
F	2 (0.62%)	0 (0%)
G	0 (0%)	0 (0%)
H	2 (0.62%)	0 (0%)
I	0 (0%)	0 (0%)

Cohort I, Pre-implementation cohort; Cohort II, Post-implementation cohort; three DRPs in cohort II concerning problem with cost efficiency not rated regarding patient outcome as no medication error as per definition.

#### 3.1.5 Rates of unsolved DRPs at hospital discharge

**Figure 3:**
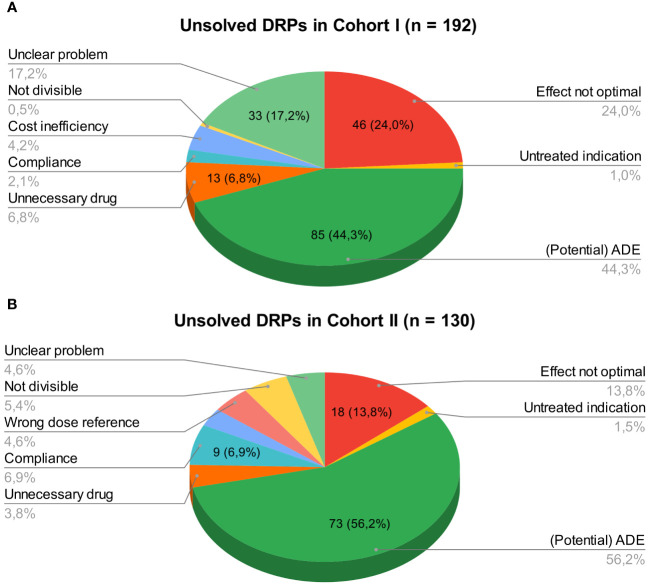
**(A)** Unsolved DRPs at discharge in cohort I (paper-based medication charts). **(B)** Unsolved DRPs at discharge in cohort II (digital medication charts). Unsolved DRPs at discharge **(A)** in cohort I (paper-based medication charts). **(B)** in cohort II (digital medication charts). Not divisible: Dosage form not divisible. Unnecessary drug: Unnecessary drug treatment. Compliance: Bad compliance or patient satisfaction with treatment.

**Figure 4:**
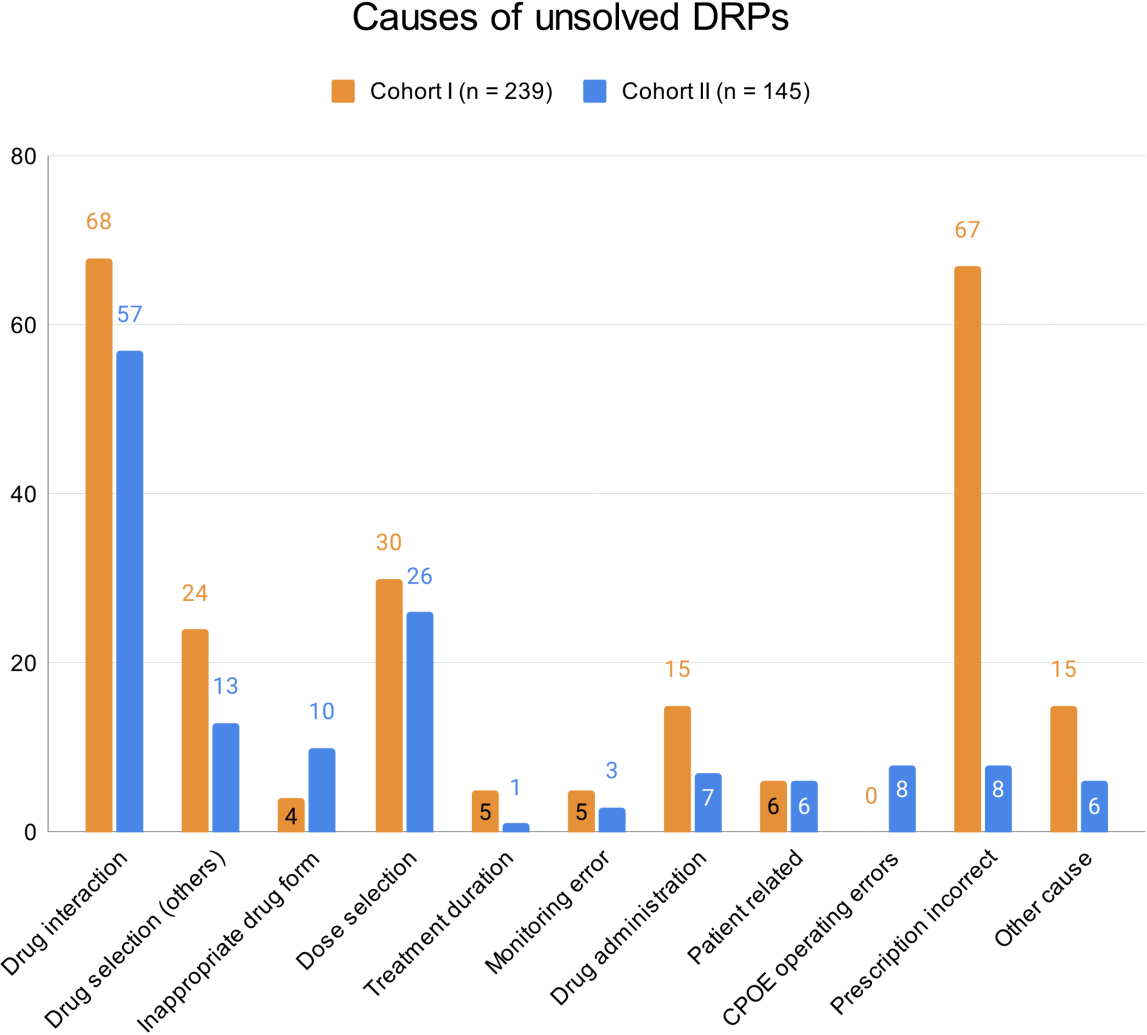
Causes of unsolved DRPs at discharge in cohorts I and II. Drug selection (others): DRPs caused by drug selection excluding drug interactions. More than one cause per DRP possible.

**Figure 5:**
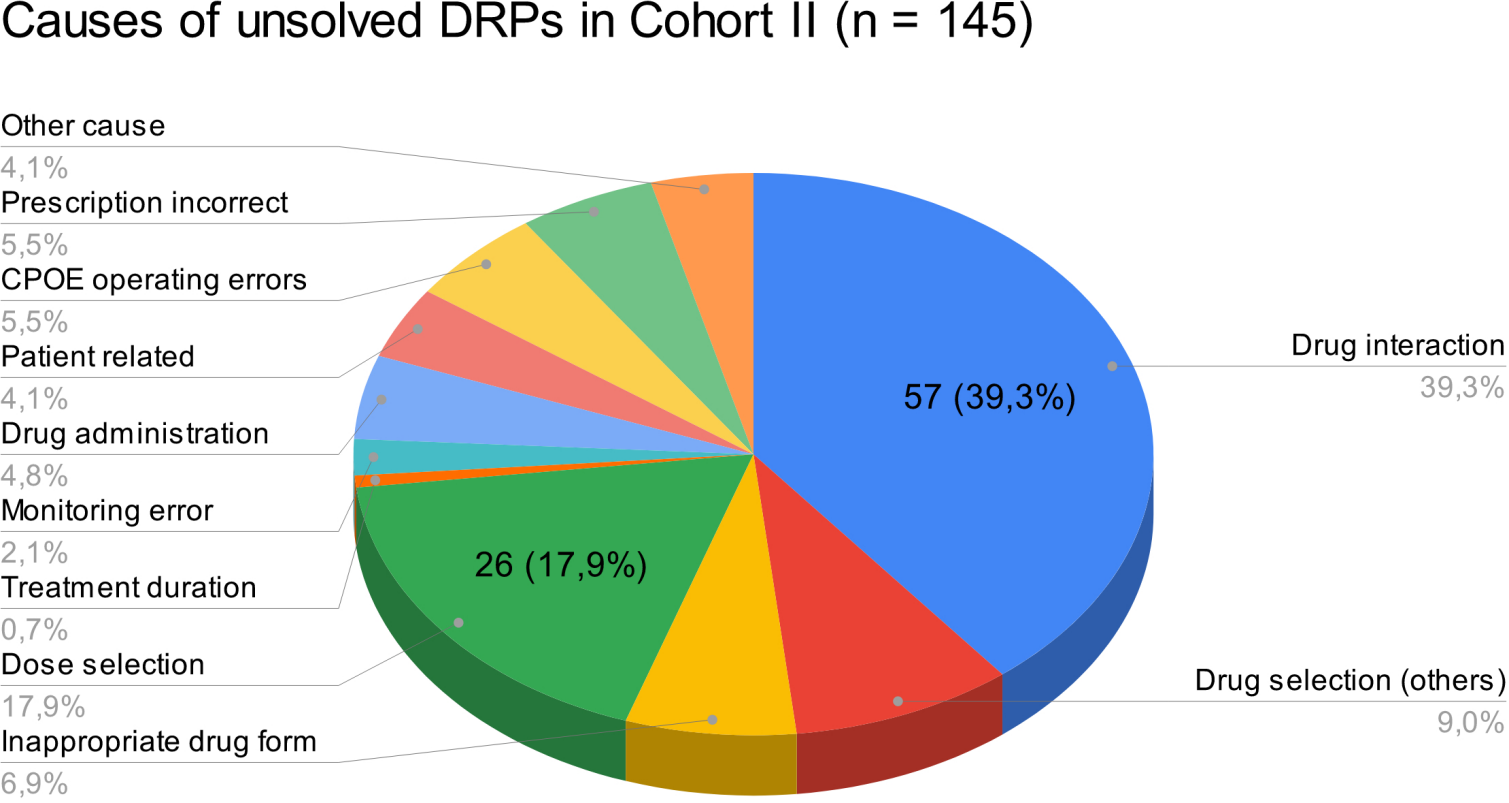
Causes of unsolved DRPs at discharge in cohort II. Drug selection (others): DRPs caused by drug selection excluding drug interactions. More than one cause per DRP possible.

At hospital discharge, 3.6 (SD 2.9) and 2 (SD 2.1) DRPs per patient remained unsolved in cohort I and in cohort II, respectively (OR=0.573, 95% CI 0.415-0.793, p*<*0.001, Pearson’s r=-0.298). The risk for a DRP increased significantly per year of age (OR=1.017, 95% CI 1.007-1.028, p=0.001). Of all unsolved problems, 2.9 (SD 2.7) DRPs per patient in cohort I and 1.4 (SD 1.9) DRPs per patient in cohort II were categorized as solvable (OR for unsolved but solvable DRPs at discharge =0.481, 95% CI 0.324-0.713, p*<*0.001, Pearson’s r=-0.306). Again, the only covariate significantly effecting the odds for a DRP was patient age (OR=1.019, 95% CI 1.006-1.031, p=0.004). The types of DRPs unsolved at discharge in cohort I and II are presented in **Figures 3A, B**. The causes of DRPs unsolved at discharge are compared in **Figure 4**. **Figure 5** illustrates the causes of unsolved DRPs in cohort II after the intervention. Out of 57 unsolved DRPs caused by drug interactions after CPOE implementation, 21 (36.8%) drug combinations increased the risk for prolongation of the patients’ QT-intervals.

#### 3.1.6 Drugs involved in DRPs

A total of 140 drugs were involved in the DRPs composed of 97 drugs in 506 drug prescriptions in cohort I and 91 drugs in 335 drug prescriptions in cohort II. The drug most frequently involved in DRPs in both cohorts was quetiapine (cohort I: n=37 (involved in 11.4% of all DRPs; 7.3% of all documented drugs); cohort II: n=28 (involved in 13.1% of all DRPs; 8.4% of all documented drugs); p=0.599), the most frequently involved drug class was antidepressants (N06A; cohort I: n=164 (involved in 50.5% of all DRPs; 32.4% of all documented drugs); cohort II: n=129 (involved in 60.3% of all DRPs; 38.5% of all documented drugs); p=0.076). The most frequently involved non-psychotropic ATC group in both cohorts was R06A (antihistamines for systemic use; 35/506, 6.9% in cohort I and 15/335, 4.5% in cohort II) with promethazine as the most common representative drug (34/506, 6.7% in cohort I and 12/335, 3.6% in cohort II) followed by M01A (antiinflammatory and antirheumatic products, non-steroids; 22/506, 4.3% in cohort I and 9/335, 2.7%) with incomplete PRN prescriptions as a common cause for DRPs. Within the ATC group M01A, the most frequently involved drug was ibuprofen (19/506, 3.8% in cohort I and 8/335, 2.4% in cohort II).

Overall, psychotropic drugs (N05, N06) accounted for 63.0% and 60.3% of all drugs involved in DRPs in cohorts I and II, respectively. **Supplementary Table S6** in the **Supplementary Material** gives an overview of the ten most frequent ATC-groups and all prescribed drugs within these groups which were involved in DRPs in both cohorts.

The dosage form most frequently involved in DRPs in both cohorts was tablet (66.2% in cohort I and 63.3% in cohort II, p=0.417, **Supplementary Table S7** in **Supplementary Material**). Most medications involved in DRPs were oral solid dosage forms (tablets, capsules, tablets and capsules with extended release, orally dissolving tablets; cohort I: 425/506, 84.0%; cohort II: 305/335, 91.0%). If other (unknown) dosage forms are excluded, 92.8% (425/458) and 95.6% (305/319) were oral solid dosage forms. 29.8% of drug prescriptions involved in DRPs were prescribed as PRN medication in cohort I (151/506) opposed to 18.2% in cohort II (61/335).

#### 3.1.7 Interventions in cohort II and their acceptance

In cohort II, 61 pharmaceutical interventions were suggested by the clinical pharmacist during the regular medication plausibility checks. 53 specific interventions were suggested to the treating physician as a written note in the digital medication chart (24.8% of all DRPs). Seven interventions (3.4%) were discussed with the prescriber via phone calls. 33 (54.1%) of all interventions suggested by the clinical pharmacist were accepted and fully implemented. 59 DRPs (27.6%) were not addressed by the clinical pharmacist during the daily medication plausibility checks but identified and documented retrospectively when screening the physician’s letters and case notes in the digital patient records and remained unsolved at discharge. 24 of these DRPs were classified as manifest problems. Only one of these DRPs needed a medical intervention as it caused temporary harm to the patient (midday tiredness after taking Pregabaline in the mornings, NCC MERP patient outcome: E).

### 3.2 Employee satisfaction survey after CPOE implementation

In total, 35 online questionnaires were received: 11 by physicians (11/35, return of 31% of all physicians working on the wards or day units at the psychiatric clinic), and 24 by nurses (24/121, return of 19.8% of all nurses working on the wards or day units at the psychiatric clinic). Overall, nurses and physicians perceived the CPOE system with the integrated CDSS as very useful (Mdn=1, IQR=1; with 1: very useful, 5: not useful at all) and labor-saving (Mdn=1, IQR=1; with 1: full agreement, 5: full disagreement). Meona^®^ fulfilled the physicians’ expectations towards a digital medication chart (Mdn=1, IQR=0; with 1: full agreement, 5: full disagreement). The documentation tools of the drug dispensing (Mdn=2, IQR=1; with 1: full agreement, 5: full disagreement) and administration processes (Mdn=1, IQR=1; with 1: full agreement, 5: full disagreement) mostly fulfilled the nurses’ expectations. On average, nurses and physicians felt competent in the use of the CPOE system (Mdn=2, IQR=1; with 1: full agreement, 5: full disagreement). Overall, the participants expected a slight decrease in the prevalence of MEs in the medication process after implementation of Meona^®^ (Mdn=4, IQR=2; with 1: significant increase, 5: significant decrease). The cooperation with the hospital pharmacy was rated consistently positive.

## 4 Discussion

To our knowledge, this is the first study that systematically assessed the types and prevalence of DRPs, including unsolved DRPs at hospital discharge, before and after CPOE implementation accompanied by regular pharmacist-led plausibility checks in psychiatric inpatients. We identified a significant decrease in the prevalence of DRPs after CPOE implementation, mostly due to the reduction of prescription errors. The prevalence of other types of DRPs was not significantly changed. Furthermore, we observed a significant risk reduction for unsolved DRPs at hospital discharge, especially regarding resolvable DRPs. This evidence supports our initial hypothesis that the implementation of a CPOE system with integrated CDSS accompanied by the introduction of regular pharmaceutical validation reduce the prevalence of DRPs and contribute to their resolution in psychiatric inpatients.

### 4.1 Rates and types of DRPs

In accordance with the World Health Organization’s Global Patient Safety Challenge *Medication Without Harm* (37), the aim of this study was to identify DRPs which limit the safety of medication practices at a psychiatric hospital in Germany. The results of our study showed that the implementation of a CPOE system with an integrated CDSS and the regular plausibility checks by a hospital pharmacist contributed to the decrease in the occurrence of DRPs from 151.9 to 81.3 DRPs per 1000 patient days.

In comparison to our results, Wolf et al. (26) found 63.0 to 87.1 DRPs per 1000 patient days prior to their implementation of a structured, interdisciplinary medicines management in psychiatric inpatients. They did, however, not include prescription errors such as missing dosage forms or missing dose instructions for PRN medication in the overall number of DRPs. As prescription errors accounted for a large proportion of DRPs in cohort I in our study (34.8%), it is plausible that we identified more DRPs. Furthermore, a longer hospital stay was significantly associated with a higher number of DRPs per patient in our cohorts. In our study, patients had longer lengths of stay in hospital (39.6 ± 20.3 and 40.5 ± 23.4) than in the study of Wolf et al. (26) (29.0-35.0 days, interquartile range: 19.8-49.0). This contributes to explaining a relatively higher prevalence of DRPs in our study cohorts. In our post-implementation cohort, only 5.6% of all DRPs were caused by prescription errors which shows that overall, the prevalence of DRPs in our study cohorts was comparable to the results of Wolf et al. (26).

With regard to the types of DRPs including medication errors, a systematic review of CPOE and CDSS by Velez-Díaz-Pallarés et al. (21) reported a 71% overall reduction of prescription errors (relative risk [RR], 0.29 [95% CI, 0.10, 0.85]; I2 = 99%) after CPOE implementation. However, there were no significant differences in rates of validation, dispensing, and administration errors with CPOE versus manual prescribing (21). In our study, we did not calculate a RR for prescription errors before and after CPOE implementation as we did not document the total number of drug prescriptions per patient throughout hospitalization. Nonetheless, we measured an OR of 0.545 (95% CI 0.412-0.721) for DRPs after CPOE implementation with a large reduction of incomplete or erroneous prescriptions from n=113 (34.8% of all DRPs, mean=2.1 per patient) in cohort I to n=12 (5.6% of all DRPs, mean=0.2 per patient) in cohort II. If we calculated an estimated RR based on the TDB at admission, it would be RR=0.09 (95% CI 0.05-0.15, number needed to treat: 2.49, 95% CI 2.17-2.93) for CPOE implementation. However, there were more total drug prescriptions than can be calculated by the TDB at admission.

Velez-Díaz-Pallarés et al. (21) also reported an increase of certain medication errors such as duplication errors. Furthermore, Brown et al. (38) identified eight key themes associated with the introduction of new types of CPOE-related prescription errors in primary and secondary care: computer screen display, drop-down menus and auto-population, wording, default settings, non-intuitive ordering or information transmission, repeat prescriptions and automated processes, users work processes, and CDSS. In line with these new error types, we identified the selection of the wrong dose reference in a drop down menu during the CPOE-based prescription process as a new prescribing error with potential for harm (NCC MERP patient outcome category A).

We measured potential patient harm by classifying the patient outcome for each DRP based on the NCC MERP taxonomy (33). The most frequent outcome were circumstances with capacity to cause error (37.5% and 33.2% in pre- and post-implementation groups). Wolf et al. (26) rated 25.5% of DRPs in the control group and 25.0% in the intervention group as having little or no potential for harm which is an outcome comparable to the NCC MERP category A. Furthermore, a potential ADE (26) can be compared with the NCC MERP category D: error reached the patient, monitoring or intervention required to preclude harm. In this category, we grouped 24.6% and 29.0% of DRPs in the pre- and post-implementation groups whereas Wolf et al. (26) identified 60.1% and 58.3% in the control and intervention groups.

In contrast to Wolf et al. (26), we did not differentiate between preventable errors causing ADEs and non-preventable ADEs in the outcome classification. However, we rated the severity of the error based on the patient outcome while Wolf et al. (26) estimated the relevance of the DRPs as minor, moderate or major. A total of 38 DRPs in 54 patients (11.7% of all DRPs, 0.70 per patient) resulted in patient harm in the pre-implementation cohort in our study which is similar to a total of 14.3% and 16.7% of DRPs that resulted in actual ADEs in the control and intervention groups in Wolf et al. (26). In our post-implementation group, 27 DRPs in 65 patients (12.6% of all DRPs, 0.42 per patient) leading to temporary harm to the patient and requiring an intervention were identified. While we found two DRPs (0.6%) with temporary harm that required prolonged hospitalization in two different patients and two DRPs (0.6%) necessitating an intervention to sustain life in one patient in the pre-implementation group, we did not identify any DRPs with these severities after CPOE implementation. If we compare the absolute frequencies, we found less DRPs resulting in at least temporary harm to the patient in our cohort II after CPOE implementation. However, our cohort size was not big enough to show a significant difference. Larger studies are needed to prove a significant reduction of manifest ADEs after implementation of a CPOE system with an integrated CDSS accompanied by pharmacist validation of the medication charts.

When comparing our data to the results in the study of Wolf et al. (26), it has to be kept in mind that our study designs differed substantially: while Wolf et al. (26) used a prospective design and focused on the implementation of a structured, interdisciplinary medicines management with a pharmacist on the psychiatric wards, including a follow-up three months after discharge, we used a retrospective approach with the main intervention being the CPOE implementation with an integrated CDSS without follow-up. An effective intervention with the purpose of improving medication safety should reduce the number of unsolved DRPs at discharge to a minimum. In our study, we found more DRPs per patient during hospitalization in both control and intervention cohorts compared to Wolf et al. (26) (6.0 ± 4.7 and 3.3 ± 3.2 vs. 3.1 ± 2.6 and 3.0 ± 2.7). Additionally, more DRPs remained unsolved per patient in our study (3.6 ± 2.9 in the pre-implementation group and 2.0 ± 2.1 in the post-implementation group) than in the study of Wolf et al. (26) (2.3 ± 2.1 in the control group and 0.4 ± 0.9 in the intervention group). 49.4 DRPs per 1000 patient days were not resolved in the post-implementation group in our study compared with 5.8 unsolved DRPs in the intervention patients in the study of Wolf et al. (26). Therefore, it can be concluded that simple medication plausibility checks by hospital pharmacists are not as effective in solving DRPs as a comprehensive interdisciplinary medicines management.

Nonetheless, the implementation of a CPOE system with CDSS and pharmaceutical validation by a hospital pharmacist showed a medium effect size of Pearson’s r *>* 0.3 for the reduction of DRPs (r=-0.326) and an almost medium effect size of r=0.298 for the reduction of unsolved DRPs (39).

Similar to our study design, Hernandez et al. (22) conducted a study on CPOE implementation at an orthopedic surgery unit where a pharmacist routinely checked all drug prescriptions after CPOE implementation. They found a significant decrease in prescribing errors from 30.1% to 2.4% of all drug prescriptions (p*<*0.0001) after CPOE implementation but did not report on the number of pharmaceutical interventions contributing to the error reduction.

In our study, patient age and length of hospital stay significantly increased the risk for DRPs. Both factors were also identified as potential risk factors of DRPs in a recently published systematic review (40). We did not find a significant difference for gender while female gender was one of the identified potential risk factors in the systematic review (40). Another risk factor was at least one hospitalization in the preceding year. As the study periods of the pre- and post-implementation cohorts in our study where within 1.5 years, a higher mean count of hospital stays in the pre-implementation group (1.7 ± 1.5 vs. 1.4 ± 1) might have resulted in a higher risk for DRPs. However, the mean count of hospital stays was not significantly different between the two groups (p=0.14). An increased number of medications is another risk factor for DRPs (40). Although many different definitions of the term polypharmacy exist, the most common one is the use of five or more medications daily (41). Following this definition, with a mean TDB of 4.8 (SD 3) and 4.8 (SD 3.6), on average, neither the patients in the pre-implementation cohort nor in the post-implementation cohort were exposed to polypharmacy on their second inpatient day.

Similar to the study results of Wolf et al. (26), psychotropic drugs accounted for more than half of all drugs involved in DRPs in our study and the most frequently involved drug was quetiapine. In contrast to Wolf et al. (26), we did not document all prescribed drugs but only those involved in DRPs. Therefore, we were not able to calculate the potential to cause a DRP per prescribed drug. Antidepressants were involved in more than half of all identified DRPs in our study. Alshaikhmubarak et al. (40) also identified antidepressants and antipsychotics as risky medications for DRPs among others. We would like to point out another group of drug prescriptions which was involved in numerous DRPs in our study: the PRN medication. 72 out of 442 drugs (16.3%) involved in DRPs where the patient had to be monitored or an intervention was required to prevent harm were prescribed as PRN with 16.8% (44/262) in cohort I and 15.6% (28/180) in cohort II. We advise physicians to always take into account which drugs have already been prescribed as PRN and to keep the combinations within this section at a minimum.

Another aim of this study was to assess the risk for anticholinergic ADEs in psychiatric inpatients using the TDB_AC_, ACB and DBI_AC_ scores. For patients aged ≥ 65 years, whenever feasible, a reduction of their ACB score sum to under 3 has been recommended (42). In both study cohorts in our retrospective study, the mean ACB score was >2 (3 ± 2 in cohort I and 2.4 ± 1.4 in cohort II) which suggests that on average, patients were exposed to a clinically relevant ACB. Furthermore, in older persons, an increase of DBI by one unit has been associated with worse overall physical performance, usual gait speed and grip strength after five years (43). A high DBI has usually been defined as ≥ 1 and is associated with a decreased overall physical performance, usual gait speed, grip strength, and Barthel index and an increased risk of hospital admission due to delirium in older people aged ≥ 65 years (43–45). Few studies have studied the effects of an increased anticholinergic burden in patients younger than 65 years. In a cohort of 106 clinically stable patients with schizophrenia with a mean age of 39.9 years (SD 11.3), a higher anticholinergic load was associated with a decrease in attention and memory (46). The patients in our study were on average 4.6 to 6.1 years older (46.0 ± 14.9 in cohort I and 44.5 ± 17.0 in cohort II) and mostly had a diagnosis of depression (79.6% vs. 76.9%).

In our study, we only included anticholinergic but not sedative drugs in the DBI calculation. Nonetheless, with a mean DBI_AC_ higher than 1, even without sedative drugs, the patients in our cohorts were exposed to a high anticholinergic load. Examples of adverse anticholinergic effects are dryness of mouth, dizziness, constipation, urinary retention, agitation, confusion, memory impairment, and tremor (47). As some of these effects do not directly harm patients but might reduce their quality of life, patients with mental health conditions who are exposed to anticholinergic drugs for most of their lifetime should be monitored for anticholinergic side effects. However, only few anticholinergic ADEs such as urinary retention, dryness of mouth and constipation were found among the patients in our study, e.g. urinary retention in a 58-year old woman with an ACB-score of 10 and DBI_AC_=3.78. In total, anticholinergic ADEs were documented in six patients in the pre-implementation and in two patients in the post-implementation cohorts. We therefore suggest that monitoring of anticholinergic drug burden might not be as clinically relevant in depressed patients younger than 50 years.

### 4.2 Pharmaceutical interventions after CPOE implementation and their acceptance

Previous studies indicated that the utilization of a CDSS may improve communication and knowledge about drug therapy among staff and therefore increases the number of accepted clinical pharmacy interventions (48). In their study, Calloway et al. (48) found an increase from 1986 documented accepted interventions per month by clinical pharmacy staff prior to CDSS implementation to 4065 accepted interventions per month post CDSS implementation. The authors estimated that cost savings related to pharmacist interventions increased from an average of $127,467 per month before to $249,959 per month after CDSS introduction, representing a 96% increase in cost savings per year (48).

Compared to the study of Wolf et al. (26), fewer but still more than half of all pharmaceutical interventions were accepted by the ward staff in our study (65.6% vs. 88.6%). A systematic review on the impact of clinical pharmacist interventions in patients with mental health disorders summarized data from 15 studies including 1986 patients with psychiatric illness with a mean age of 54.5 ± 22.4 years (49). Inappropriate drug selection and ADEs were the most common DRPs identified in the included studies (49). In all referenced studies combined, 2714 out of 3611 pharmaceutical interventions (75.1% ± 20.4%, range: 29-100%) were accepted and lead to a change in drug therapy (49). The acceptance rate in our study was within the range of those found in the review.

In another study assessing pharmaceutical care in a long-stay psychiatric hospital, 60.3% of all DRPs with known outcome remained unsolved whereof 34.2% were partially solved and for the other 65.8%, there was either no need or no possibility for resolution (50). In our study, after CPOE implementation, 30% of all unsolved DRPs did not have to be solved or were unsolvable and 9.2% were partially solved. Almost half (45.4%) of the unsolved DRPs in our study were only identified retrospectively during the extensive study of the patient files and medication charts but not addressed during the daily medication plausibility checks by the pharmacist. 63.1% of all DRPs with known outcome remained unsolved whereas 36.9% were completely solved.

Almost half of all DRPs which remained unsolved in the post-implementation group were at least partly caused by drug selection (48.3%), most being drug interactions (57/145, 39.3%). However, not all drug combinations causing drug interactions are preventable in psychiatric inpatients and not all drug interactions are actually clinically harmful to the patients. In our study, only 28 out of all 179 DRPs (15.6%) caused by DDIs resulted in manifest problems such as QT-prolongation >450 ms (9/78 DDIs, 11.5% in cohort II).

Additionally, when discussing acceptance rates and patient outcome concerning DRPs after CPOE and CDSS implementation, the phenomenon of CDSS alert fatigue has to be considered. If the CDSS in a CPOE system generates multiple alerts during the drug prescription process, they might be ignored by the prescribing physician (51). As we did not assess the rates of CDSS acceptance and overriding in our study, it is possible that CDSS alert fatigue might have decreased the effect of the pharmaceutical interventions in our study which were mostly written notes to the physicians.

Following a different approach, Hahn et al. (52) validated the pharmacist-physician collaboration in psychiatry in ‘the Eichberger-model’. In this model, a clinical pharmacist is employed directly in a psychiatric hospital in Germany providing drug interaction checks, patient counseling and drug information to physicians (52). 82.1% of all physicians employed at the psychiatric hospital during the study period sent e-mail requests regarding drug therapy optimization to the clinical pharmacist, most frequently regarding appropriate drug selection, drug-drug interactions and ADEs (52). Each of the pharmaceutical recommendations was accepted and 98.6% were implemented by the physicians (52).

To sum up, these results suggest that a more patient-oriented approach with a pharmacist on the ward as part of an interdisciplinary team facilitates the resolution of a higher proportion of solvable DRPs during hospitalization.

### 4.3 Validity assessment

We calculated an overall concordance of 98% [range: 96-100% for the different categories] between the ratings of the two pharmacists with the final classification of the DRPs. This concordance level was higher than in Ihbe-Heffinger et al. (34) but might have been over-estimated as only two raters were involved in our study compared to 37 in Ihbe-Heffinger et al. (34). Furthermore, in our study, the second pharmacist was not blinded to the DRP classifications of the first pharmacist and the ratings were therefore not independent.

### 4.4 Employee satisfaction survey

The nurses and physicians responding to the employee satisfaction survey one year after CPOE implementation were mostly satisfied with the implementation process from training and implementation to working with the software on the wards. They also anticipated the decrease of MEs through CPOE implementation. In line with these expectations, the number of DRPs per patient and per 1000 patient days was significantly lower after CPOE implementation. Nonetheless, operating errors of the medication software were documented in 19 cases (8.9% of DRPs after CPOE implementation). The most common error of choosing the wrong dose reference in the drop-down menus during the prescription process was addressed in further training sessions by pharmacists after the study period. We expect that most of the remaining prescription errors are currently corrected by the validating pharmacist on the first day after hospital admission and therefore do not reach the patient.

### 4.5 Strengths and limitations

In our retrospective study, we assessed DRPs in psychiatric inpatients in a real world setting. Neither the prescribing physicians nor the hospital pharmacist conducting the medication plausibility checks knew that a patient treated at the time would be included in the study later on. Therefore, our results have a high external validity as they reflect the actual DRPs which occurred during the regular medication process in psychiatric inpatients. Another strength of this study was that it involved only low personnel resources. Therefore, similar projects of CPOE implementation and an accompanying introduction of medication plausibility checks by a clinical pharmacist can surely be realized in other psychiatric hospitals without the resources to install a pharmacist on every ward.

However, there are several limitations to this study. The retrospective study included data from a time period during the COVID-19 pandemic. Some patients included in the study, were discharged earlier from the psychiatric ward due to an infection with SARS-CoV-2 or because they had close contact with someone who tested positive for COVID-19. Therefore, the average length of hospital stay per patient might have been lower in the study than outside the COVID-19 pandemic.

Additionally, we only calculated the number of drugs prescribed once for each patient on the first full inpatient day. Medications and their dosages are often changed during the relatively long stay of psychiatric inpatients. Therefore, we cannot rule out that different prescribing patterns during hospitalization might have led to differences in the number of drugs prescribed between the two cohorts. However, we chose the first full inpatient day for the score calculations because the number of DRPs per patient increases per additional drug prescribed on admission (10).

Another limitation is that the classification of the same DRPs may be different when other documentation systems are used as Wolf et al. (53) reported for the two German documentation systems PIE (54) and DokuPIK (32). Wolf et al. (53) especially criticized the lack of detail in the DokuPIK classification resulting in insufficient information on the type of DRP when used for study purposes. To address this concern, the classification used in our study was based on the validated PCNE (13) and NCC MERP classifications (30) and used more detail than the DokuPIK database.

Furthermore, the research team was unblinded to the group allocation of patients to the pre- or post-implementation cohorts. As we expected fewer DRPs after CPOE implementation and more resolved DRPs after the introduction of medication validation by a pharmacist, it is possible that we searched for DRPs more carefully on the paper-based medication charts. Nonetheless, we documented DRPs for 54 patients in the post-implementation cohort before analyzing the paper-based medication charts. All DRPs were documented by the same pharmacist first before being checked by the second pharmacist. As usual in our clinical routine, only pharmacists were involved in the identification and categorization of DRPs. Psychiatrists might have a different view about the clinical relevance of DRPs. However, all manifest DRPs documented in the CPOE-cohort were discussed with a senior psychiatrist who agreed to the importance of addressing these problems in clinical practice. Therefore, we appraise the identification and documentation of DRPs to be complete and comparable between both groups.

As an additional limitation, we did not include missing indications in drug prescriptions, especially in PRN medication, as a potential prescribing error. Therefore, if this specific type of error would be considered, the error rates are expected to be substantially higher in both groups.

Moreover, due to the retrospective design of the study, DRPs could only be identified if they were documented by the ward staff during the patients’ hospitalization. As a consequence, the actual number of DRPs might have been higher in both groups if patients did not report ADEs to the ward staff or if documentation was incomplete. Furthermore, no medication reconciliation was completed by a pharmacist and prior medication plans were seldom available in the electronic patient records. Therefore, transcription errors at admission were not identifiable and might be under-reported.

In spite of the limitations, as we reached a high concordance level in the categorization, our results have a high internal validity. The medication process from digital prescription to pharmaceutical validation can therefore serve as an example for other psychiatric hospitals with an implemented CPOE system.

### 4.6 Conclusion

DRPs occur frequently in psychiatric hospitals and present a potential threat to patient safety. Few studies have examined the impact of pharmacist-led clinical interventions such as medication reviews in psychiatric inpatient settings. However, no study has been published on the effect of the implementation of a CPOE system with CDSS combined with medication plausibility checks by clinical pharmacists on the rate of identified DRPs during hospital stay and unsolved DRPs in psychiatric patients at hospital discharge.

This retrospective study indicated that the implementation of a CPOE system with CDSS combined with medication plausibility checks by a clinical pharmacist may reduce the number of DRPs in psychiatric inpatients and contribute to solving them during the patient’s hospital stay. While the design did not allow to draw a direct link between intervention and outcomes, the study identified categories of DRPs and responsible drug classes that are most likely to have the potential to cause harm in psychiatric inpatients. Drug-drug interactions were identified as one major DRP with the potential to lead to adverse events in the study cohorts. Further research is needed on tailored clinical interventions to solve the most dangerous DRPs in psychiatric practice. It should aim at creating clinical practice guidelines to assist psychiatrists in creating a safe medication process for every patient in mental health settings.

## Data availability statement

The raw data supporting the conclusions of this article will be made available by the authors, without undue reservation.

## Ethics statement

The studies involving humans were approved by Ethikkommission der Universität zu Lübeck. The studies were conducted in accordance with the local legislation and institutional requirements. The participants provided their written informed consent to participate in this study.

## Author contributions

KW: Writing – original draft, Visualization, Methodology, Investigation, Formal Analysis, Data curation, Conceptualization. JT: Writing – review & editing, Supervision, Project administration, Methodology, Conceptualization. AN: Writing – review & editing, Validation, Investigation. B-LM: Writing – review & editing, Validation, Supervision. SB: Writing – review & editing, Validation, Supervision, Project administration, Methodology.
