# Supplementary material for: Reduced prevalence of drug-related problems in psychiatric inpatients after implementation of a pharmacist-supported computerized physician order entry system - a retrospective cohort study
Source: Front Psychiatry. 2024 Apr 9;15:1304844. doi: 10.3389/fpsyt.2024.1304844 (PMC11035719; doi:10.3389/fpsyt.2024.1304844)
Supplement: Supplementary file 1 [file DataSheet_1.pdf]

Supplementary Material

1 SUPPLEMENTARY DATA

The datasets for this study are available upon request from the study authors.

2 SUPPLEMENTARY TABLES AND FIGURES

Table S1. Information sources for CDSS in Meona®
[a]SmPC: Summary of product characteristics
[b]BfArM: German Federal Institute for Drugs and Medical Devices
[c]EMA: European Medicines Agency
[d]FDA: US Food and Drug Administration

**Source**	**Content**
The ABDA database by ABDATA/AVOXA	information on drugs, medicinal product agreements, interactions with morbidities, allergies, life circumstances (e.g. pregnancy)
Preston, C.: Stockley's Drug Interactions	drug interactions
SmPCs[a], package leaflets	legal information on medicinal product by manufacturer, incl. dosage adjustments in renal impairment
'Rote-Hand-Briefe' (transl.: 'Red-Hand-Letters')	official letters on newly identified, clinically relevant risks of specific drugs and actions for risk minimization by pharmaceutical companies published by BfArM[b], German equivalent to FDA[d] black box warnings
Guidelines by professional societies	recommendations for e.g. choice of drugs, dosages, treatment duration
Information by BfArM[b], EMA[c], FDA[d]	legal information by health authorities
Renal Drug Handbook	dosage adjustments in renal impairment
NICE British National Formulary (BNF)	dosage adjustments in renal impairment

Table S2. Classification of DRPs and Patient Outcome based on The PCNE Classification for Drug-Related Problems V9.1, the NCC MERP Taxonomy of Medication Errors and Doku-PIK. Some categories were added by the study team (IDM-PSY-PHARM). Additionally, problems were rated as potential or manifest problems. Cat.: Category

Category	Primary domain	Subdomain	Source	Code
Problems	Treatment effectiveness	No effect of drug despite correct use	PCNE	P1.1
		Effect of drug treatment not optimal	PCNE	P1.2
		Untreated symptoms or indication	PCNE	P1.3
	Treatment safety	Adverse drug event (possibly) occurring	PCNE	P2.1
	Other	Unnecessary drug-treatment	PCNE	P3.1
		Bad patient compliance/satisfaction	IDM-PSY-PHARM	
		Problem with cost efficiency	IDM-PSY-PHARM	
		Wrong dose reference (salt or base form)	IDM-PSY-PHARM	
		Dosage form not divisible	IDM-PSY-PHARM	
		Unclear problem/complaint	PCNE	P3.2
Causes	Drug selection	Inappropriate drug	PCNE	C1.1
		No indication	PCNE	C1.2
		Inappropriate combination (drug interaction)	PCNE	C1.3
		Inappropriate duplication of therapeutic group or active ingredient	PCNE	C1.4
		Insufficient drug treatment in spite of indication	PCNE	C1.5
		Too many drugs for indication	PCNE	C1.6
		Not listed in hospital's list of medicines	IDM-PSY-PHARM	
	Drug form	Inappropriate drug form	PCNE	C2.1
	Dose selection	Drug dose too low	PCNE	C3.1
		Drug dose too high	PCNE	C3.2
		Dosage regimen not frequent enough	PCNE	C3.3
		Dosage regimen too frequent	PCNE	C3.4
		Dose timing instructions wrong, unclear or missing	PCNE	C3.5
	Treatment duration	Duration of treatment too short	PCNE	C4.1

Category	Primary domain	Subdomain	Source	Code
		Duration of treatment too long	PCNE	C4.2
	Monitoring Error	Documented Allergy	NCC MERP	70.12.3
		Drug-Disease Interaction	NCC MERP	70.12.4
		Clinical (e.g. blood glucose, blood pressure)	NCC MERP	70.12.5
	Drug administration by health professional	Inappropriate timing of administration or dosing intervals	PCNE	C6.1
		Drug under-administered	PCNE	C6.2
		Drug over-administered	PCNE	C6.3
		Drug not administered	PCNE	C6.4
		Wrong drug administered	PCNE	C6.5
		Drug administration via wrong route	PCNE	C6.6
		Administration not documented	IDM-PSY-PHARM	
	Patient related	Patient intentionally uses/takes less than prescribed or no drug at all	PCNE	C7.1
		Patient uses/takes more drug than prescribed	PCNE	C7.2
		Patient abuses drug (unregulated overuse)	PCNE	C7.3
		Patient decides to use unnecessary drug	PCNE	C7.4
		Patient takes food that interacts	PCNE	C7.5
	Other	No or inappropiate outcome monitoring	PCNE	C9.1
		Other cause	PCNE	C9.2
		No obvious cause	PCNE	C9.3
		Operating errors of medication software	IDM-PSY-PHARM	
		Transcription error	NCC MERP	87.7
		Prescription/Documentation incomplete/incorrect	Doku-PIK	
Planned Interventions	No intervention	No intervention	PCNE	I0.1
	At prescriber level	Prescriber informed only	PCNE	I1.1
		Prescriber asked for information	PCNE	I1.2
		Intervention proposed to prescriber	PCNE	I1.3
		Intervention discussed with prescriber	PCNE	I1.4

Category	Primary domain	Subdomain	Source	Code
	At drug level	Drug changed to . . .	PCNE	I3.1
		Dosage changed to . . .	PCNE	I3.2
		Formulation changed to . . .	PCNE	I3.3
		Instructions for use changed to . . .	PCNE	I3.4
		Drug paused or stopped	PCNE	I3.5
		Drug started	PCNE	I3.6
		Time of intake changed	IDM-PSY-PHARM	
	Other	TDM or laboratory control suggested	Doku-PIK	
		Assistance with drug procurement	Doku-PIK	
		Side effect reported to authorities	PCNE	I4.2
		Other intervention (see comments)	IDM-PSY-PHARM	
Intervention Acceptance	Intervention accepted	Intervention accepted, fully implemented	PCNE	A1.1
		Intervention accepted, partially implemented	PCNE	A1.2
		Intervention accepted but not implemented	PCNE	A1.3
		Intervention accepted, implementation unknown	PCNE	A1.4
	Intervention not accepted	Intervention not accepted: not feasible	PCNE	A2.1
		Intervention not accepted: no agreement	PCNE	A2.2
		Intervention not accepted: other reason (specify)	PCNE	A2.3
		Intervention not accepted: unknown reason	PCNE	A2.4
	Other	Intervention proposed, acceptance unknown	PCNE	A3.1
		Intervention not proposed	PCNE	A3.2
Status of the DRP	Not known	Problem status unknown	PCNE	O0.1
	Solved	Problem totally solved	PCNE	O1.1
	Partially solved	Problem partially solved	PCNE	O2.1
	Not solved	Problem not solved, lack of cooperation of patient	PCNE	O3.1
		Problem not solved, lack of cooperation of prescriber	PCNE	O3.2

Category	Primary domain	Subdomain	Source	Code
		Problem not solved, intervention not effective	PCNE	O3.3
		Problem not solved, detected retrospectively	IDM-PSY-PHARM	
		No need or possibility to solve problem	PCNE	O3.4
Patient Outcome	No Error	A: Circumstances with capacity to cause error	NCC MERP	31.1
	Error, No Harm	B: Error did not reach patient	NCC MERP	31.1
		C: Error reached patient, medication administered	NCC MERP	31.1
		C: Error reached patient, medication not administered	NCC MERP	31.1
		D: Error reached patient, monitoring or intervention required to preclude harm	NCC MERP	31.1
	Error, Harm	E: Error with temporary harm, intervention required	NCC MERP	33.1
		F: Error with temporary harm, initial or prolonged hospitalization required	NCC MERP	33.2
		G: Error with permanent harm	NCC MERP	33.3
		H: Error required intervention to sustain life	NCC MERP	33.4
	Error, Death	I: Error contributed to/resulted in patient's death	NCC MERP	34.1

Table S3. Patient diagnoses as ICD-10 codes (World Health Organization, 2019) in the study groups before and after CPOE implementation
Cohort I: Pre-implementation cohort; Cohort II: Post-implementation cohort

**Ranking of diagnoses**	**Diagnoses cohort I**<br>No. of patients out of n=54 (%)	**Diagnoses cohort II**<br>No. of patients out of n=65 (%)
1	F33.2<br>33 (61%)	F33.2<br>32 (49%)
2	F42.2<br>11 (20%)	F32.2<br>11 (17%)
3	F40.01<br>7 (13%)	F42.2<br>8 (12%)
4	F34.1<br>7 (13%)	F40.1<br>7 (11%)
5	F41.1<br>5 (9%)	F40.01<br>7 (11%)
6	F43.1<br>5 (9%)	F34.1<br>6 (9%)
7	60.31<br>5 (9%)	F41.1<br>5 (8%)
8	F32.2<br>4 (7%)	F43.1<br>5 (8%)
9	F60.6<br>4 (7%)	F10.2<br>4 (6%)
10	U07.2!<br>4 (7%)	F42.0, F41.0, F90.0 and F33.1<br>3 each (5%)

Table S4. Examples of manifest and potential DRPs in cohort I before CPOE implementation

[a]: For patient outcome categories refer to Table S2; TDM[b]: Therapeutic drug monitoring; PRN[c]: pro re nata, medication as needed; *QT interval was not prolonged in ECG controls.

Examples of manifest and potential DRPs in Cohort I	Description	DRP	Cause	Patient Outcome[a]
Manifest	Patient reported micturition difficulties and mouth dryness after taking combination of quetiapine and escitalopram.	ADR	Drug-drug interaction	E
	Amplification of antidopaminergic effects, increased risk for extrapyramidal symptomps and neuroleptic malignant syndrome by combining melperone, promethazine, olanzapine, lithium, and quetiapine	ADR	Drug-drug interaction	H
	Amplification of sedative and respiratory depressive effects by combining melperone, olanzapine, lithium, and lorazepam	ADR	Drug-drug interaction	H
	Patient experienced urinary retention with several anticholinergic drugs prescribed: amisulprid, olanzapine, lorazepam, venlafaxine, doxepine (ACB-score: 10, DBI_{AC}: 3.78)	ADR	Drug-drug interaction	E
	Ibuprofen prescribed up to four times 400 mg per day, without defining the dosage form	Potential ADE	Incomplete prescription	A
	Dose reduction of lithium at admission due to high dose (2x 900 mg/d) without prior TDM[b]: Patient felt hypomanic after dose reduction	Effect of drug treatment not optimal	Drug dose too low	E
	Massive sleep disturbance without adequate response to mirtazapine in a smoker: mirtazapine is metabolised faster by smokers.	Effect of drug treatment not optimal	No TDM/Drug interaction	F
	Promethazine prescribed 4x 25 mg PRN[c] without dosage form	Unclear problem	Incomplete prescription	A

Examples for manifest and potential DRPs in Cohort I	Description	DRP	Cause	Patient Outcome[a]
	Haloperidol prescribed 5 mg PRN[c] without dosage form	Unclear problem	Incomplete prescription	A
	Dose administered higher than the prescribed dose (e.g. doxepine 50 mg administered by nurse, only 25 mg prescribed)	ADR	Drug over-administered by a health professional	E
Potential	QT-prolongation: Multiple drugs prescribed that can lead to prolongation of the patient's QT-interval (e.g. quetiapine, escitalopram, promethazine).	ADR	Drug-drug interaction	D*
	Ibuprofen and candesartan may increase plasma levels of lithium	Potential ADR	Drug-drug interaction	D
	Combination of escitalopram and doxepine increases risk for serotonine syndrome	Potential ADR	Drug-drug interaction	D
	Bupropione may increase plasma levels of citalopram	Potential ADR	Drug-drug interaction	D
	Fluoxetine inhibits the metabolic enzyme CYP2D6 and can therefore increase plasma levels of e.g. risperidone, amitriptyline	Potential ADR	Drug-drug interaction	D
	Chlorprothixene may diminish the dopaminergic effects of levodopa	Effect of drug treatment not optimal	Drug-drug interaction	D
	Pantoprazole prescribed PRN, inappropriate drug choice as PRN	Effect of drug treatment not optimal	No indication for drug	A
	Quetiapine prescribed without dosage form: film-coated tablets and prolonged-release tablets available	Effect of drug treatment not optimal	Incomplete prescription	A
	Incomplete prescription of self medication in medication chart (e.g. folic acid, inhalator)	Unclear problem	Incomplete prescription	A

Examples for manifest and potential DRPs in Cohort I	Description	DRP	Cause	Patient Outcome[a]
	Deviating information on PRN medication in medication chart and discharge letter	Unclear problem	Erroneous prescription at transfer	A

Table S5. Examples of manifest and potential DRPs in cohort II after CPOE implementation

[a]: For patient outcome categories refer to Table S2; $D^{!}$: QT interval was prolonged in ECG controls; D*: QT interval was not prolonged in ECG controls; C^{1}: C, medication administered.

Examples of manifest and potential DRPs in Cohort II	Description	DRP	Cause	Patient Outcome[a]
Manifest	QT-prolongation: Multiple drugs prescribed that can lead to prolongation of the patient's QT-interval (e.g. venlafaxine and prothipendyl; venlafaxine, amitriptyline and dimenhydrinate; opipramole and doxepine).	ADR	Drug-drug interaction	$D^{!}$
	Patient reported gastrointestinal complaints after first intake of venlafaxine	ADR	Other cause: Possible ADR of Venlafaxine at regular doses	E
	Patient is very tired in the morning after taking quetiapine 25 mg tablet at bedtime (22:00) for three days	ADR	Dosage too high for patient	E
	Fluoxetine inhibits the metabolic enzyme CYP2D6 and can therefore increase plasma levels of aripiprazole: Patient reported severe restlessness and difficulties falling asleep	ADR	Drug-drug interaction	E
	Patient reported to be tired during the day after taking clomipramine in the mornings. Clomipramine should be taken in the evening or at bedtime.	Bad patient compliance/satisfaction	Dose timing instructions wrong	E
	Patient experienced amenorrhoea under olanzapine therapy, laboratory results showed hyperprolactinaemia	ADR	No obvious cause	E
	Melatonine prolonged-release tablets prescribed as PRN for insomnia. They should be prescribed daily 1-2 h before bedtime, inappropriate as PRN.	Effect of drug treatment not optimal	Dosage regimen not frequent enough	C^{1}

Examples of manifest and potential DRPs in Cohort II	Description	DRP	Cause	Patient Outcome[a]
	No effect of amitriptyline 50 mg per day in a smoking patient, plasma level not analysed. Amitriptyline can be metabolised faster in smokers.	No effect of drug	Drug interaction/Drug dose too low	E
	Dominal®forte 80 mg tablets prescribed as 40 mg per intake, tablet cannot be divided	Drug cannot be divided according to manufacturer labelling	Division of a non-dividable drug prescribed	A
	Venlafaxine prescribed as salt (225 mg venlafaxine hydrochloride), but dose per prolonged-release capsule refers to venlafaxine base	Wrong salt or base form prescribed	Operating error of medication software	A
Potential	QT-prolongation: Multiple drugs prescribed that can lead to prolongation of the patient's QT-interval (e.g. amitriptyline, aripiprazole, promethazine).	Potential ADR	Drug-drug interaction	D*
	Entresto®only prescribed once daily but should be taken twice daily	Effect of drug treatment not optimal	Dosage regimen not frequent enough	C[1]
	Clomipramine newly prescribed in a patient on treatment with 20 mg citalopram. Advised against overlapping intake due to increased risk for serotonine syndrome.	Potential ADR	Drug-drug interaction	C[1]
	In patients aged over 65 years, the maximum dose for escitalopram is 10 mg. 20 mg prescribed in a patient aged over 65 years without TDM.	Potential ADR	Drug dose too high	D
	Mirtazapine prescribed against insomnia in a strong smoker. Mirtazapine can be metabolised faster in smokers.	Effect of drug treatment not optimal	Other cause: Drug interaction with smoking status	A
	Two drops (containing 2 mg of Promethazine) of Atosil®20 mg/ml prescribed, insufficient effect of drug treatment expected.	Effect of drug treatment not optimal	Drug dose too low	D

Examples of manifest and potential DRPs in Cohort II	Description	DRP	Cause	Patient Outcome[a]
	Oxycodone prolonged-release tablets prescribed as PRN, immediate-release tablets would be more appropriate.	Effect of drug treatment not optimal	Inappropriate drug form	B
	Combination of high doses of oxycodone/naloxone and chlorprothixene: amplification of sedative and hypotensive effects, respiratory depression possible	Potential ADR	Drug-drug interaction	A
	Tramadole als PRN medication increases risk for serotonine syndrome unter drug therapy with venlafaxine	Potential ADR	Drug-drug interaction	A
	Quetiapine 200 mg immediate-release tablet prescribed at bedtime (22:00) in a depressed patient as the only antidepressive drug, prolonged-release tablet would be more appropriate (intake in the evening)	Effect of drug treatment not optimal	Inappropriate drug form	A

Table S6. Absolute and relative frequencies of the top ten ATC-classes (WHO Collaborating Centre, 2023) and all drugs prescribed within which were involved in DRPs in the study groups before and after CPOE implementation
Cohort I: Pre-implementation cohort; Cohort II: Post-implementation cohort

Drug or drug class	Cohort I [n=325]	Cohort II [n=214]
**A02B**	**18 (5.5%)**	**8 (3.7%)**
Esomeprazole	2 (0.6%)	0 (0%)
Omeprazole	1 (0.3%)	4 (1.9%)
Pantoprazole	15 (4.6%)	4 (1.9%)
**C07A**	**8 (2.5%)**	**4 (1.9%)**
Bisoprolol	1 (0.3%)	1 (0.5%)
Sotalol	0 (0%)	1 (0.5%)
Metoprolol	7 (2.2%)	2 (0.9%)
**M01A**	**22 (6.8%)**	**9 (4.2%)**
Etoricoxib	0 (0%)	1 (0.5%)
Ibuprofene	19 (5.8%)	8 (3.7%)
Diclofenac	2 (0.6%)	0 (0%)
Naproxene	1 (0.3%)	0 (0%)
**N02B**	**8 (2.5%)**	**2 (0.9%)**
Acetylsalicylic acid combination	0 (0%)	1 (0.5%)
Paracetamol	5 (1.5%)	0 (0%)
Metamizole	3 (0.9%)	1 (0.5%)
**N04B**	**8 (2.5%)**	**1 (0.5%)**
Levodopa/Benserazid	8 (2.5%)	1 (0.5%)
**N05A**	**119 (36.6%)**	**61 (28.5%)**
Amisulpride	9 (2.8%)	1 (0.5%)
Aripiprazole	16 (4.9%)	12 (5.6%)
Chlorprothixene	8 (2.5%)	4 (1.9%)
Haloperidol	5 (1.5%)	0 (0%)
Lithium	15 (4.6%)	4 (1.9%)
Olanzapine	8 (2.5%)	4 (1.9%)
Pipamperone	4 (1.2%)	1 (0.5%)
Prothipendyl	8 (2.5%)	2 (0.9%)
Melperone	5 (1.5%)	4 (1.9%)
Quetiapine	37 (11.4%)	28 (13.1%)
Risperidone	4 (1.2%)	1 (0.5%)
**N05B**	**25 (7.7%)**	**4 (1.9%)**
Benzodiazepine (not specified)	0 (0%)	1 (0.5%)
Diazepam	3 (0.9%)	0 (0%)
Lorazepam	21 (6.5%)	3 (1.4%)
Lavandulae aetheroleum	1 (0.3%)	0 (0%)
**N05C**	**9 (2.8%)**	**5 (2.3%)**
Melatonin	0 (0%)	1 (0.5%)

Valerianae radix	3 (0.9%)	0 (0%)
Zolpidem	0 (0%)	2 (0.9%)
Zopiclone	6 (1.8%)	2 (0.9%)
**N06A**	**164 (50.5%)**	**129 (60.3%)**
Agomelatin	2 (0.6%)	0 (0%)
Amitriptyline	14 (4.3%)	20 (9.3%)
Citalopram	4 (1.2%)	4 (1.9%)
Clomipramine	0 (0%)	7 (3.3%)
Doxepine	11 (3.4%)	3 (1.4%)
Bupropione	6 (1.8%)	6 (2.8%)
Escitalopram	29 (8.9%)	17 (7.9%)
Fluoxetine	11 (3.4%)	0 (0%)
Fluvoxamine	0 (0%)	1 (0.5%)
Milnacipran	0 (0%)	2 (0.9%)
Mirtazapine	34 (10.5%)	18 (8.4%)
Opipramole	0 (0%)	2 (0.9%)
Sertraline	15 (4.6%)	27 (12.6%)
Tranylcypromine	1 (0.3%)	0 (0%)
Trimipramine	11 (3.4%)	2 (0.9%)
Venlafaxine	26 (8%)	20 (9.3%)
**R06A**	**35 (10.8%)**	**15 (7.0%)**
Promethazine	34 (10.5%)	12 (5.6%)
Desloratadine	1 (0.3%)	0 (0%)
Dimenhydrinate	0 (0%)	3 (1.4%)

Table S7. Absolute and relative frequencies of dosage forms involved in DRPs in the study groups before and after CPOE implementation
Cohort I: Pre-implementation cohort; Cohort II: Post-implementation cohort; Other*: Dosage form not named in prescription; [a]: Fisher's exact test; n.c.: not calculated

**Dosage form**	**Cohort I** [n=506]	**Cohort II** [n=335]	**p-value**
Tablet	335 (66.2%)	212 (63.3%)	0.417[a]
Other*	48 (9.5%)	16 (4.8%)	n.c.
Tablet (extended release)	39 (7.7%)	52 (15.5%)	n.c.
Capsule (extended release)	31 (6.1%)	23 (6.9%)	n.c.
Capsule	19 (3.8%)	11 (3.3%)	n.c.
Oral liquid	15 (3.0%)	5 (1.5%)	n.c.
Creams/Ointments/Gels/Pastes	7 (1.4%)	6 (1.8%)	n.c.
Inhalatives	7 (1.4%)	1 (0.3%)	n.c.
Injectables	4 (0.8%)	2 (0.6%)	n.c.
Orally dissolving tablet	1 (0.2%)	7 (2.1%)	n.c.

REFERENCES

World Health Organization. International Statistical Classification of Diseases and Related Health Problems, 10th Revision (2019). `https://icd.who.int/browse10/2019/en` [Accessed: 2023-05-12].

WHO Collaborating Centre. ATC/DDD Index 2023 (2023). `https://www.whocc.no/atc_ddd_index/` [Accessed: 2023-05-12].
